# A small‐molecule screen identifies novel aging modulators by targeting 5‐HT/DA signaling pathway

**DOI:** 10.1111/acel.14411

**Published:** 2024-11-18

**Authors:** Shi‐Wei Ye, Shuang‐Di Song, Xi‐Juan Liu, Yun Luo, Shi‐Qing Cai

**Affiliations:** ^1^ Institute of Neuroscience and State key Laboratory of Neuroscience, CAS Center for Excellence in Brain Science and Intelligence Technology Chinese Academy of Sciences Shanghai China; ^2^ University of Chinese Academy of Sciences Beijing China

**Keywords:** Calmagite, Carbamazepine, DAF‐16, excitatory/inhibitory balance, healthy aging, *numr‐1/−2*

## Abstract

The risk of many human diseases including cardiovascular diseases, cancer, neurodegenerative diseases, and musculoskeletal disorders rises significantly in the elderly. With the increase in the aging population, it is becoming increasingly important to understand the biology of healthy aging and develop interventions that slow down the aging process or prevent age‐related diseases. In this study, by a high‐throughput screen in *Caenorhabditis elegans* (*C. elegans*), we identified 11 small molecules that promote healthy aging. Among them, Carbamazepine (a voltage‐gated channels inhibitor) and Calmagite (a calcium and magnesium indicator) enhanced serotonin (5‐HT) and dopamine (DA) levels, extended lifespan, and preserved several important behaviors in aging *C. elegans*. These behaviors include slowing responses to food, pharyngeal pumping, locomotion, and male mating. Interestingly, we further found that administration of Carbamazepine or Calmagite alleviated hyperexcitability of aging male diagonal muscles and improved behavioral performance by ameliorating Ca^2+^ homeostasis. Mechanistically, administration of Carbamazepine or Calmagite induced nuclear translocation of the transcription factor DAF‐16 and thus up‐regulated its downstream genes *numr‐1/−2*, which are known to promote resistance to metal‐induced stresses and longevity. Taken together, our study offers a way for the discovery of drugs that promote healthy aging, and provides potential interventions for preventing behavioral deterioration in the elderly.

## Graphical abstract

Through a small‐molecule screen in *C. elegans* for aging modulators, we identified two compounds Carbamazepine and Calmagite, which elevated the 5‐HT/DA level and ameliorated age‐related behavioral decline. Carbamazepine and Calmagite prevented aging phenotypes partially by ameliorating Ca^2+^ homeostasis of excitable cells in aging worms via the transcription factor DAF‐16 and its downstream genes *numr‐1/−2*. Thus, our study offers a way for the discovery of aging modulators, and provides potential interventions for preventing behavioral deterioration in the elderly.

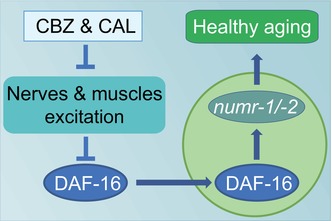

**Abbreviations**
5‐HT: serotonin
BAS‐1: DOPA decarboxylase
BSR: basal slowing response
*C. elegans*
: *Caenorhabditis elegans*
CAL: Calmagite
CBZ: Carbamazepine
DA: dopamine
DEGs: differentially expressed genes
ESR: enhanced slowing response
GABA: γ‐aminobutyric acid
IIS: insulin/insulin‐like growth factor signaling
RNAi: RNA interference

## 1 INTRODUCTION

In past decades, scientists have found some interventions that increase lifespan in animal models, including genetic manipulation, drug administration, and dietary restriction (Campisi et al., 2019; Guarente, 2014; M. Hansen & Kennedy, 2016). However, recent studies in mice, flies, and worms have shown that lifespan could be decoupled with healthspan, and extension of lifespan does not always slow down the rate of aging (Bansal et al., 2015; Guarente, 2014; M. Hansen & Kennedy, 2016; Yin et al., 2014, 2017). Thus, the biology of health aging emerges as a hot topic in the field of aging research. However, finding ways to prevent functional decline and achieve healthy aging is still a challenging task.

The nervous system coordinates the aging rate of the entire body, affects the functional decline of other tissues, and ultimately regulates healthspan. Age‐related changes in the nervous system include a decrease in the number of synapses, reduced neurotransmitter production, and impaired neural plasticity (Jin & Cai, 2023; Mattson & Arumugam, 2018). These changes result in behavioral deterioration, including impairment of cognitive function, decrease of muscle strength and motor coordination, and diminishment of sensory function (Liu et al., 2013; Toth et al., 2012; Wirak et al., 2022). Alteration of neurotransmitter signaling, which has been consistently observed in the aging nervous systems (J. Y. Hansen et al., 2022; Makman & Stefano, 1993), contributes to behavioral deterioration associated with the aging process (Braskie et al., 2008; Costa et al., 2023; Wong et al., 1984). For example, the decreased level of DA, a neurotransmitter involved in motivation and reward, leads to altered reward system and decreased working memory ability in older adults (Ciampa et al., 2022; Costa et al., 2023). Similarly, reduction in acetylcholine, a neurotransmitter involved in learning and memory, contributes to age‐related cognitive decline (Lee & Kim, 2022). Reduced γ‐aminobutyric acid (GABA) transmission alters excitatory/inhibitory balance in aging brains, and the GABA signaling regulates animal lifespan via multiple downstream transcription factors (Chun et al., 2015; Rozycka & Liguz‐Lecznar, 2017; Wirak et al., 2022).

Our previous studies have demonstrated that the levels of neurotransmitters 5‐HT and DA decrease with age, resulting in deterioration of some important behaviors in *Caenorhabditis elegans* (Yin et al., 2014). Age‐related reduction in the expression level of *C. elegans* DOPA decarboxylase (named BAS‐1, the shared 5‐HT‐ and DA‐synthesizing enzyme) is responsible for the loss of these neurotransmitters and behavioral deterioration. By using GFP‐fused BAS‐1 as a genetically traceable maker of aging, we previously performed a genome‐wide RNA‐interference‐based screen and had successfully identified conserved aging modulators (Yuan et al., 2020). In this study, using a similar screening system, we performed a small‐molecule screen for compounds that promote healthy aging. 11 hits with various bioactivities were identified. Two of them, Carbamazepine (CBZ) and Calmagite (CAL) improved 5‐HT and DA levels, extended lifespan, and preserved several important behaviors by reducing cell excitability in aging worms. The effect of CBZ and CAL is dependent on DAF‐16, the key transcription factor that modulates aging and longevity. Thus, our study identifies small molecules that promote healthy aging and provides potential drugs for preventing behavioral deterioration.

## 2 RESULTS

### 2.1 A small‐molecule screen identifies novel aging modulators

**FIGURE 1:**
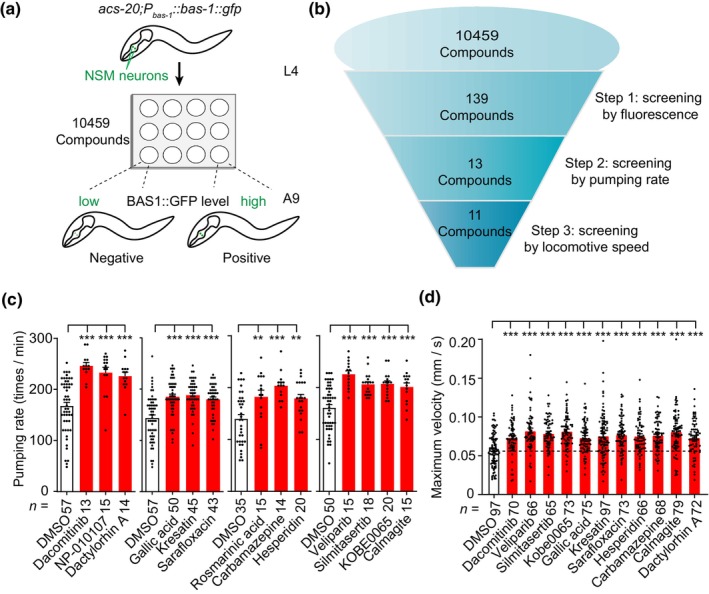
A small‐molecule screen identifies novel aging modulators. (a) The working flow of the small‐molecule screen. Compounds that increased the BAS‐1::GFP level in *acs‐20;P*_*bas‐1*_ *::bas‐1::gfp* transgenic aged worms (at day 9 of adulthood, A9) were regarded as positive hits. (b) Illustration showing the summary of the small‐molecule screen. After three rounds of screening, we obtained 11 compounds that prevented age‐related decline in the BAS‐1::GFP level, pharyngeal pumping rates, and maximum velocity in aged worms. (c) 13 compounds of the selected 55 hits with *p* value less than 0.01 were found to improve pharyngeal pumping behavior in worms at day 5 of adulthood. (d) 11 compounds were found to improve maximum velocity of worms at day 7 of adulthood. For (c), (d), the numbers of tested worms are shown beneath the bars. Data shown are means ± s.e.m.; *p < 0.05; **p < 0.01; ***p < 0.001 (one‐way ANOVA test).

Previously, by using GFP‐fused BAS‐1 as a traceable marker of aging, we performed a genome‐wide RNA‐interference (RNAi) screen in *C. elegans* and had identified novel genes that regulate age‐related behavioral deterioration (Yuan et al., 2020). We assumed that the same system could also be used to screen for small molecules that promote healthy aging. In order to increase penetration of drugs into worms, we crossed *acs‐20* null mutant worms, which exhibit defects in the cuticle barrier (Kage‐Nakadai et al., 2010), with the transgenic worms (named *P*_*bas‐1*_ *::bas‐1::gfp*) that express GFP fused BAS‐1. We then obtained *acs‐20;P*_*bas‐1*_ *::bas‐1::gfp* worms, and used them for drug screening according to the strategy depicted in Figure 1a. We firstly screened more than 10,000 small molecules from three compound libraries (Table S1) and obtained 139 screening hits that enhanced BAS‐1::GFP fluorescence level in aged *acs‐20;P*_*bas‐1*_ *::bas‐1::gfp* worms at day 9 of adulthood. We then selected 55 hits, which up‐regulated BAS‐1::GFP level at a *p* value less than 0.01 (Table S2), for the secondary round of testing by examining pharyngeal pumping rates in aged worms, and at this round we got 13 hits (Figure 1b,c and Figure S1a). Previous research has shown that maximum velocity declines with age and correlates well with longevity in *C. elegans* (Hahm et al., 2015). Next, we examined the effect of these 13 hits on maximum velocity in aged worms and found that 11 out of 13 candidates enhanced maximum velocity of aged worms (Figure 1b,d, Figure S1b and Table 1). Not surprisingly, these 11 compounds show various bioactivities: Dacomitinib, Veliparib, Silmitasertib, Kobe0065, and Gallic acid have antitumor activity; Gallic acid, Kresatin, Sarafloxacin, Dactylorhin A, and Hesperidin are anti‐inflammatory agents; Hesperidin shows antioxidant activity; CBZ is a blocker of voltage‐gated channels (Ambrosio et al., 1999); and CAL is a calcium and magnesium indicator (Rasouli & Ghavami, 2016). Alteration in neuronal Ca^2+^ homeostasis plays an important role in the process of aging (Celsi et al., 2009), and the compounds CBZ and CAL may regulate Ca^2+^ homeostasis in cells. We therefore selected CBZ and CAL for further validation.

**TABLE 1:** 11 compounds that enhance pharyngeal pumping rates and maximum velocity in aging worms.

Compound	Description	Bioactivity
Dacomitinib (M2‐G9)	Dacomitinib is a specific and irreversible inhibitor of the ERBB family (Engelman et al., 2007)	Antitumor activity
Veliparib (M31‐B3)	Veliparib is a potent inhibitor of PARPs (Donawho et al., 2007)	Antitumor activity
Silmitasertib (M50‐E10)	Silmitasertib is a highly selective and potent CK2 inhibitor (Siddiqui‐Jain et al., 2010)	Antitumor activity
Kobe0065 (M51‐B7)	Kobe0065 is a novel and effective inhibitor of Ras–Raf interaction (Shima et al., 2013)	Antitumor activity
Gallic acid (M14‐E4)	Gallic acid is a free radical scavenger to inhibit COX‐2 with antimicrobial activity (Amaravani et al., 2012; Bak et al., 2013)	Anti‐inflammatory, antitumor activity
Kresatin (T14‐G11)	Kresatin is a pharmaceutical intermediate and antiseptic (DiFiore et al., 1983)	Anti‐inflammatory activity
Dactylorhin A (A46‐C5)	Dactylorhin A exhibits inhibitory effect on NO production effects in macrophage cells (Zhao et al., 2018)	Anti‐inflammatory activity
Sarafloxacin (T12‐E4)	Sarafloxacin is a fluoroquinolone antibiotic registered (McConville et al., 1995)	Anti‐inflammatory activity
Hesperidin (M50‐D9)	Hesperidin is an anti‐inflammatory agent and exerts antioxidant effects (Tejada et al., 2018)	Anti‐inflammatory, antioxidant activity
Carbamazepine (M13‐F9)	Carbamazepine is a voltage‐gated channel blocker and used to prevent seizures (Willow & Catterall, 1982)	Regulating cell excitability
Calmagite (M50‐A4)	Calmagite is a complexometric indicator for detecting calcium and magnesium (Rasouli & Ghavami, 2016)	Regulating cell excitability

### 2.2 CBZ and CAL improve behavioral performance in aging worms

**FIGURE 2:**
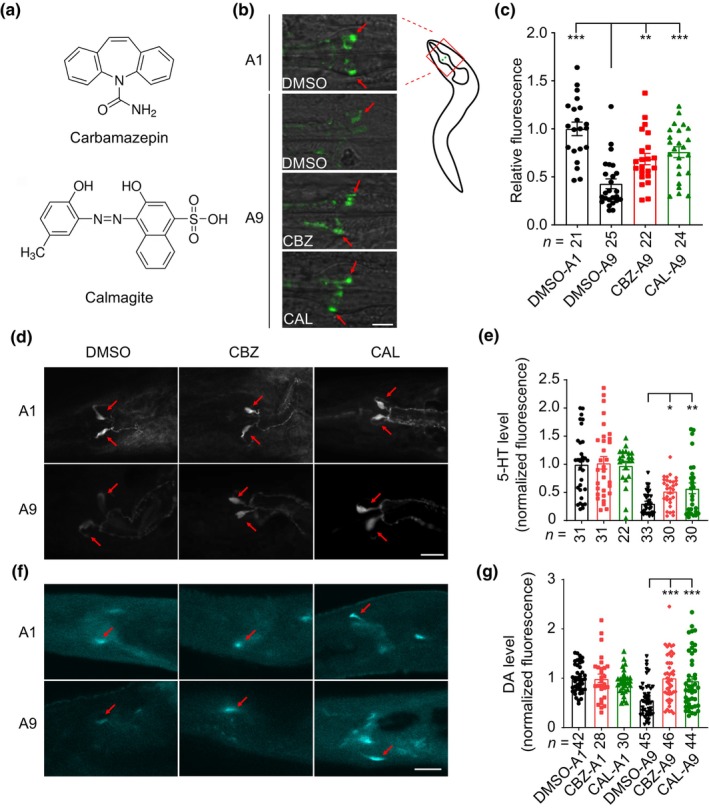
CBZ and CAL increase 5‐HT and DA levels in aging worms. (a) Molecular formulas of CBZ and CAL. (b) Fluorescent images and (c) quantitative analysis of BAS‐1 expression levels in NSM neurons in the absence or presence of CBZ or CAL. Quantitative analysis of BAS‐1 protein levels was performed by measuring GFP fluorescence intensity. Red arrows indicate the NSM neurons. (d) 5‐HT immunostaining of NSM neurons in young adult (A1) and aged worms at day 9 of adulthood (A9) in the presence of DMSO, CBZ or CAL. Red arrows indicate the NSM neurons. (e) Quantitative analysis of 5‐HT levels in NSM neurons of young adult and aged N2 worms. (f) FIF staining of CEP neurons in young adult and aged worms treated with DMSO, CBZ and CAL. Red arrows indicate the CEP neurons. (g) Quantitative analysis of DA levels in CEP neurons of young adult and aged N2 worms. For b, d and f, normalized fluorescence was obtained by dividing individual value with average fluorescence intensity of DMSO‐treated A1 worms. Scale bar, 10 μm. For (c), (e), and (g), the numbers of tested worms are shown beneath the bars. Data shown are mean ± s.e.m. *p < 0.05; **p < 0.01; ***p < 0.001 (one‐way ANOVA test).

We found that administration of CBZ and CAL significantly enhanced the expression level of BAS‐1 in aged worms (Figure 2a–c). In line with this, the neuronal level of 5‐HT, which was examined by immunostaining using anti‐5‐HT antibodies, decreased in aged worms; and administration of CBZ or CAL markedly up‐regulated 5‐HT level in aged, but not young adult worms (Figure 2d,e). Similarly, the neuronal level of DA measured by formaldehyde‐induced fluorescence (FIF) was also up‐regulated in an age‐dependent pattern after administration of CBZ or CAL (Figure 2f,g). Taken together, these data indicate that CBZ and CAL specifically up‐regulate 5‐HT and DA levels in aging worms. Age‐related reduction in endogenous 5‐HT and DA levels led to the decline of some important animal behaviors (Yin et al., 2014). We then asked whether 5‐HT or DA deficiency could abolish the beneficial effects of CBZ and CAL. We utilized two mutants, *tph‐1*(*mg280*) and *cat‐2*(*e1112*), which are deficient in the synthesis of 5‐HT and DA, respectively. We found that the two mutant strains exhibited lower maximum velocity in locomotive behavior at day 7 of adulthood compared to age‐matched N2 worms. Administration of CBZ or CAL failed to enhance the locomotive behavior of these two mutants (Figure S2), suggesting that 5‐HT or DA deficiency abolishes the beneficial effects of CBZ and CAL.

**FIGURE 3:**
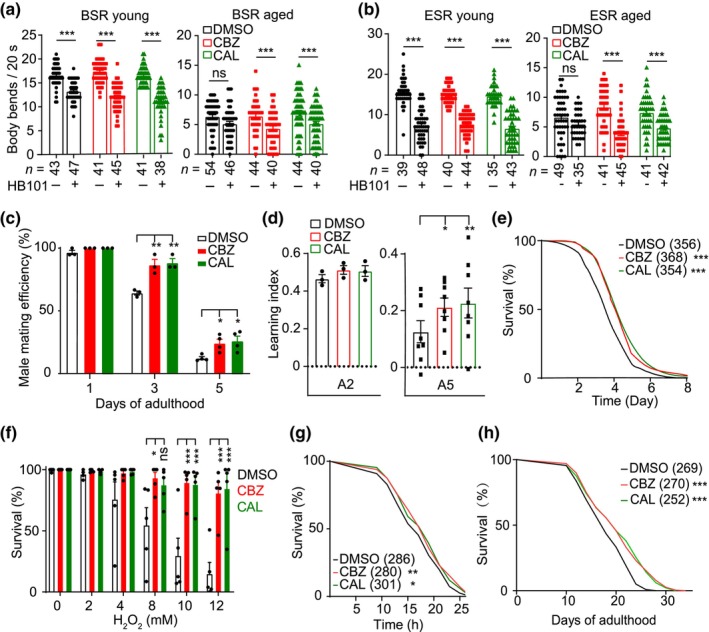
CBZ and CAL delay age‐related behavioral decline and extend lifespan. (a) BSR and (b) ESR behaviors in young adult and aged worms. BSR and ESR were quantified by the times of body bends in 20 s. The *E. coli* strain HB101 was used as worm's food. The numbers of tested worms are shown beneath the bars. (c) Mating efficiency of N2 males treated with DMSO, CBZ or CAL. (d) Learning abilities of DMSO‐, CBZ‐ or CAL‐treated worms at day 2 (A2) or day 5 (A5) of adulthood. Learning abilities were measured by testing the chemotaxis to butanone of worms immediately after a single training, which was carried out by feeding prestarved worms with OP50 in the presence of 10% butanone. Learning index = trained chemotaxis index ‐ naïve chemotaxis index. (e–g) Survival percentage of worms treated with DMSO, CBZ or CAL under ultraviolet (e), oxidative (f), or heat shock (g) stresses. (h) Lifespan curves of worms treated with DMSO, CBZ or CAL. For (a–d) and (f), the data shown are means ± s.e.m. For (e, g) and (h), data represent the sum of animals in multiple experiments. Data were from at least three independent experiments. *p < 0.05; **p < 0.01; ***p < 0.001; ns, not significant (a, b, unpaired two‐tailed Student's *t* test; c, d and f, one‐way ANOVA test; e, g and h, two‐sided log‐rank test.).

Next, we tested if CBZ or CAL could improve other behaviors in aged worms. We firstly examined whether the compounds affect food‐dependent behaviors. When encountering bacteria, well‐fed N2 worms usually reduce their locomotion speed, while food‐deprived worms dramatically enhance their slowing response. The two behaviors known as basal (BSR) and enhanced slowing response (ESR) are mediated by DA and 5‐HT, respectively (Sawin et al., 2000). Consistent with our previous results (Yin et al., 2014), we found diminished BSR and ESR in aged worms (Figure 3a,b). Interestingly, administration of CBZ or CAL preserved BSR and ESR behaviors in aged worms, but did not affect the behaviors in young adult worms (Figure 3a,b). We also found that administration of CBZ or CAL significantly alleviated age‐dependent deterioration of male mating behavior, another important behavior regulated by DA and 5‐HT (Figure 3c).

Previous studies have shown that the ability of learning and associative memory declines with age in *C. elegans* (Kauffman et al., 2011; Son et al., 2019). We then asked whether CBZ and CAL could protect the cognitive function in aging worms. *C. elegans* worms exhibit adaptive chemotaxis to butanone by pre‐exposing the worms to butanone in cultivating plates with food. We measured the learning ability of worms by subtracting the index of chemotaxis to butanone in naïve worms (before training) from the index in worms subjected to a single training, which was carried out by pre‐exposing the worms to food with 10% butanone. Consistent with previous results (Kauffman et al., 2011), the chemotaxis indexes of trained worms at day 5 of adulthood significantly reduced compared with those at day 2 of adulthood, while the chemotaxis to butanone in naïve worms remained unchanged, indicating a significantly reduction in learning ability in aging worms (Figure S3). Strikingly, we found that administration of CBZ or CAL markedly enhanced butanone appetitive learning in aged worms (Figure 3d), suggesting a protective role of CBZ and CAL in regulating cognitive aging in *C. elegans*.

Nematodes often encounter to intricate environmental stressesthat disrupt their intracellular homeostasis and physiology. The stress resistance capacity is a hallmark of their longevity and survival ability. We found that administration of CBZ or CAL significantly improved the ability of aging worms to withstand ultraviolet light, hydrogen peroxide, and 35°C heat shock (Figure 3e–g), suggesting that CBZ and CAL promote intracellular homeostasis in *C. elegans*. Expectedly, N2 worms treated with CBZ or CAL exhibited a significant extension of lifespan (Figure 3h). Taken together, these data suggest that administration of CBZ or CAL improves 5‐HT and DA function and extends healthspan in *C. elegans*.

### 2.3 CBZ and CAL affect behavioral decline via regulating cellular excitability

Some behavioral declines correlate with increased cell excitability. For instance, the male‐specific diagonal muscles that control turning behavior during mating become more excitable in aging males (Guo et al., 2012). Then, we asked whether CBZ and CAL modulate aging via regulating cell excitability. To address this issue, we used ratiometric Ca^2+^ imaging by overexpressing the Ca^2+^ indicator protein GCaMP5 together with DsRed2 (both are driven by the diagonal‐muscle‐specific *unc‐68* promoter) (Chen et al., 2015) to explore the excitability of diagonal muscles. To minimize movement, we used paralyzed *unc‐54*(*e190*) hermaphrodites as mating mates during Ca^2+^ imaging assays.

**FIGURE 4:**
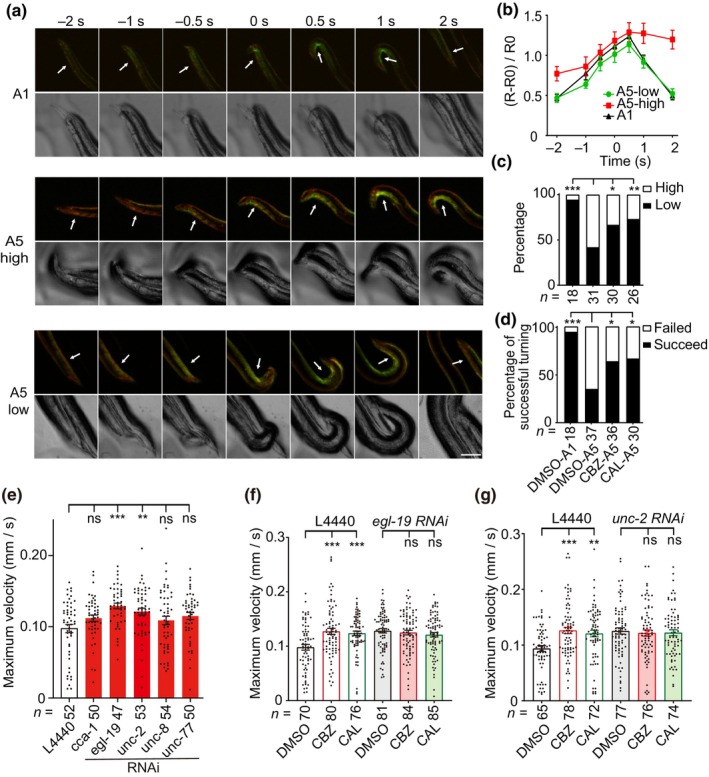
CBZ and CAL suppress the excitability of diagonal muscles in aging males. (a) GCaMP5 (green)/DsRed2 (red) fluorescent images and phase‐contrast light images of diagonal muscles of N2 males during turning behavior. Paralyzed *unc‐54*(*e190*) hermaphrodites were used as mating mates for tested males. White arrows indicate diagonal muscles. Scale bar, 20 μm. (b) Changes in ΔR/R0 at diagonal muscles of N2 males at day 1 (A1) and day 5 (A5) of adulthood during turning behavior. R means the fluorescence ratio of GCaMP5/DsRed2. The fluorescence ratio of GCaMP5/DsRed2 of a male moved alone (without a hermaphrodite) was regarded as R0. The time point of 0 represents the examined males' posterior tails reached the terminus of hermaphrodites' body (heads or tails). The value of ΔR/R0 reached maximum at time point of 0.5 s in males during turning. A5 males with the value of (ΔR/R0 at 2 s)/(ΔR/R0 at 0.5 s) ≥ 0.75 were classified as a high excitability group and otherwise as a low excitability group. Data shown are mean ± s.e.m. (c) The percentage of high and low excitability groups in DMSO‐, CBZ‐ or CAL‐treated worms. (d) Male turning efficiency in DMSO‐, CBZ‐ or CAL‐treated worms. Only the first attempt was counted for each male. (e) The maximum velocity of worms at day 7 of adulthood (A7) after down‐regulation of some Na^+^ and Ca^2+^ channels by RNAi. (f, g) The maximum velocity of A7 worms fed with control, *egl‐19* (f) or *unc‐2* (g) dsRNAs in the presence of DMSO, CBZ or CAL. The empty L4440 plasmids were used as control dsRNAs. For (c–g), the numbers of tested worms are shown beneath the bars. *p < 0.05; **p < 0.01; ***p < 0.001 (c, d, Chi‐square test; e‐g, one‐way ANOVA test).

In the diagonal muscles of young adult males, the ratio of △R/R0 (*R* represents the value of GCaMP5/DsRed2 fluorescence, and R0 is the *R* value in the free‐moving male) began to increase when their posterior tails approached hermaphrodites' heads or tails, and reached the maximum at the time point of 0.5 s when males attempted to make a turning. After that, the Ca^2+^ signal decreased quickly (Figure 4a,b). However, aged males (at day 5 of adulthood) showed either of two patterns of Ca^2+^ dynamics during mating: some aged males exhibited a Ca^2+^ dynamics pattern similar with young adult males, that is, the ratios of △R/R0 in their diagonal muscles firstly increased and then decreased fast after successful turning; the other aged males showed persistently high Ca^2+^ signal in the diagonal muscles, and could not finish the turning step (Figure 4a,b). The ratio of the first pattern to the total is about 0.35 (Figure 4c) in aged males, consistent with a turning efficiency of 35% in aged males (Figure 4d). Interestingly, administration of CBZ or CAL improved intracellular Ca^2+^ dynamics as the values of △R/R0 in most of treated males declined fast after the time points of 0.5 s, resulting in successful turning and enhanced mating efficiency in aged males (Figure 4c,d). Thus, these results suggest that CBZ and CAL modulate aging at least partially by regulating excitability of neurons and muscles.

We next down‐regulated the expression of several Na^+^ and Ca^2+^ channel subunits by RNAi to ask how the two compounds affect the Ca^2+^ dynamics and age‐related behavioral decline. We found that knock‐down of *egl‐19* (a L‐type Ca^2+^ channel) or *unc‐2* (a P/Q‐type Ca^2+^ channel), but not *cca‐1* (a T‐type Ca^2+^ channel), *unc‐77* (a Na^+^ leak channel), and *unc‐8* (an amiloride‐sensitive Na^+^ channel), improved locomotive behavior in aging worms (Figure 4e). Furthermore, down‐regulation of *egl‐19* or *unc‐2* extended the worm's lifespan, but did not further improve the beneficial effects of CBZ and CAL, suggesting that the effect of CBZ and CAL on Ca^2+^ dynamics is related to inhibition of EGL‐19 and UNC‐2 Ca^2+^ channels (Figure 4f,g and Figure S6b).

We then asked whether CBZ and CAL inhibit L‐type and P/Q‐type Ca^2+^ channel activities directly, and performed whole‐cell recording in HEK293T cells expressing human Ca^2+^ channels Cav1.2 and Cav2.1, which are orthologues of *egl‐19* and *unc‐2*, respectively. We found that CBZ significantly blocked Cav1.2 and Cav2.1 currents, but CAL did not affect the function of the two Ca^2+^ channels (Figure S4). The Ca^2+^ chelating agent CAL probably complexes with the cellular excessive Ca^2+^ to maintain Ca^2+^ homeostasis in aging worms. Taken together, these results suggest that CBZ and CAL promote healthy aging via regulating L‐type and P/Q‐type Ca^2+^ channel‐mediated Ca^2+^ dynamics.

### 2.4 CBZ and CAL promote healthy aging via DAF‐16

**FIGURE 5:**
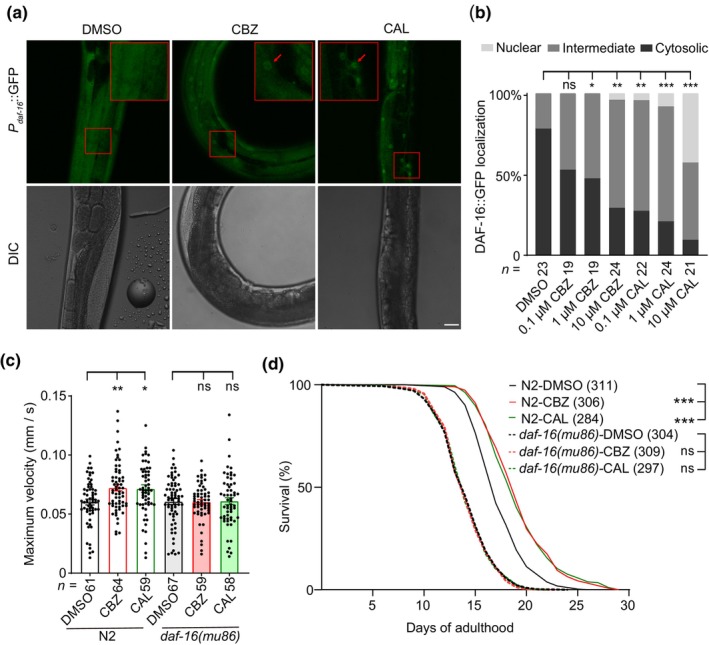
CBZ and CAL prolong healthspan via DAF‐16. (a) Fluorescent images of DAF‐16::GFP in A2 worms treated with DMSO, CBZ or CAL. Scale bar, 20 μm. (b) Quantitative analysis of DAF‐16::GFP cellular localization. (c) Age‐dependent changes of maximum velocity in N2 and *daf‐16*(*mu86*) mutant worms that treated with DMSO, CBZ or CAL. Data were from at least three independent experiments; data shown are means ± s.e.m. (d) Lifespan curves of N2 and *daf‐16*(*mu86*) mutant worms treated with DMSO, CBZ or CAL. Data represent the sum of animals in multiple experiments. For (b, c), the numbers of tested worms are shown beneath the bars. *p < 0.05; **p < 0.01; ***p < 0.001; ns, not significant (b, Chi‐square test; c, one‐way ANOVA test; d, two‐sided log‐rank test).

We next explored how CBZ and CAL modulate aging. The forkhead transcription factor DAF‐16 is the major downstream effector of the insulin/IGF‐1 signaling (IIS) pathway that controls aging and longevity (Kenyon, 2010). We then used a transgenic worm strain TJ356 (*P*_*daf‐16*_ *::daf‐16::gfp*) expressing GFP‐fused DAF‐16 (Lin et al., 2001) to investigate whether CBZ and CAL could induce nuclear translocation of DAF‐16, a key step for regulating DAF‐16 downstream genes. We found that the tested worms exhibited three different localization patterns of DAF‐16, that is, cytosolic, intermediate, and nuclear localization (Figure S5a). Most of control worms showed cytosolic localization of DAF‐16, while a much larger percentage of CBZ‐ or CAL‐treated worms exhibited intermediate and nuclear localization of DAF‐16 (Figure 5a,b), suggesting that administration of CBZ or CAL facilitates nuclear localization of DAF‐16. Consistent with this, downregulation of *egl‐19* and *unc‐2* also led to nuclear localization of DAF‐16 (Figure S6a).

We then determined whether CBZ and CAL promote healthy aging via the transcription factor DAF‐16 by knocking down *daf‐16* using RNA interference. We found that knock‐down of *daf‐16* abolished the behavioral improvement in pharyngeal pumping in aging worms treated with CBZ or CAL (Figure S5b). Furthermore, administration of CBZ or CAL did not increase the maximum velocity of locomotion behavior or extend the lifespan of *daf‐16*(*mu86*) mutant worms (Figure 5c,d), indicating that the beneficial effects of CBZ and CAL on preventing aging phenotypes are dependent on the presence of the transcription factor DAF‐16.

**FIGURE 6:**
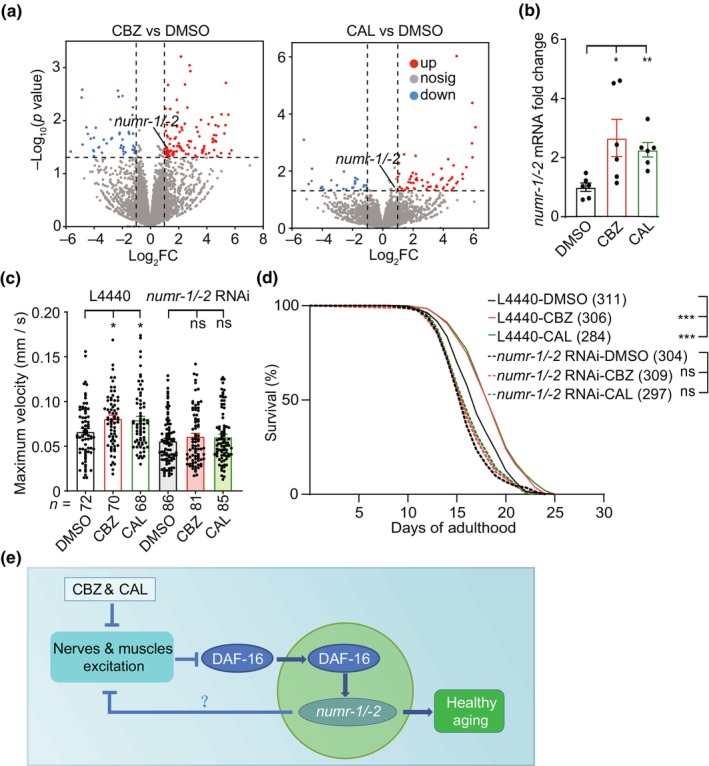
The function of CBZ and CAL is mediated by DAF‐16 downstream effectors NUMR‐1/−2. (a) Volcano plot representation of DEGs in worms treated with DMSO, CBZ or CAL. Up‐regulated and down‐regulated genes are marked in red and in blue, respectively (|log_2_FC| > 1 and FDR <0.05). (b) RT‐PCR analysis of *numr‐1/−2* gene expression levels. (c) Age‐dependent changes of maximum velocity in worms fed with control or *numr‐1/−2* dsRNAs in the presence of DMSO, CBZ or CAL. Data were from at least three independent experiments and the data shown are means ± s.e.m. (d) Lifespan curves of worms fed with control or *numr‐1/−2* dsRNAs in the presence of DMSO, CBZ or CAL. Data represent the sum of animals in multiple experiments. (e) Proposed working model for the mechanism underlying the function CBZ or CAL on modulating aging. *p < 0.05; **p < 0.01; ***p < 0.001; ns, not significant (b, c, one‐way ANOVA test; d, two‐sided log‐rank test).

We next performed RNA sequencing (RNA‐seq) assays and found that CBZ treatment induced 93 up‐regulated and 44 down‐regulated genes, while CAL treatment caused 58 up‐regulated and 28 down‐regulated genes (Figure 6a and Table S3). Only five genes were upregulated and two genes were downregulated in both CBZ‐ and CAL‐treated worms (Table S3). None of these overlapping genes has been reported to be related to IIS pathway or DAF‐16. However, we found that some IIS pathway downstream genes were significantly up‐regulated in worms treated with one of the two chemicals and showed an increasing tendency in worms treated with the other chemical (Figure S7a). Notably, by quantitative PCR analysis, we found a fraction of these genes showed significantly increased expression levels in both CBZ‐ and CAL‐ treated worms (Figure 6b and Figure S7b). The upregulated genes included *sdz‐12* (Robertson et al., 2004), *mab‐3* (Ezcurra et al., 2018), *daf‐6* (Hong et al., 2021), *numr‐1*, *numr‐2* and several collagen genes (Ewald et al., 2015) (Figure S7a). Among them, *numr‐1* and *numr‐2* are functionally equivalent genes with identical sequences and expression patterns. NUMR‐1/−2 are nuclear localized metal responsive‐1/−2 that protect animals from metal‐specific toxicity and promote longevity (Tvermoes et al., 2010). Given that the beneficial effect of CBZ and CAL on aging phenotypes is related to Ca^2+^ signal, we then asked whether the modulation of aging by CBZ and CAL is mediated by NUMR‐1 and NUMR‐2. We found that downregulation of *numr‐1/−2* by RNAi eliminated the beneficial effects of CBZ and CAL on locomotion and longevity in aging worms (Figure 6c,d).

We next generated transgenic (*P*_*numr‐1/−2*_ *::numr‐1/−2*) worms expressing NUMR‐1/−2, and found that overexpression of *numr‐1/−2* improved locomotive behavior in aging worms and extended the lifespan of worms. Furthermore, overexpression of *numr‐1/−2* did not further improve the beneficial effects of CBZ and CAL on locomotion and lifespan, suggesting that the regulation of aging by CBZ and CAL is related to NUMR‐1/−2 (Figure S8). Taken together, these data suggest that administration of CBZ or CAL induces nuclear translocation of DAF‐16, which activates downstream genes *numr‐1/−2* to promote healthy aging.

## 3 DISCUSSION

In this study, by a small‐molecule screen in *C. elegans* for drugs that promote healthy aging, we identified 11 hits with various activities. Two compounds, CBZ and CAL elevated the 5‐HT/DA level and ameliorated age‐related decline in behaviors, including pharyngeal pumping, male mating, locomotion, and food‐induced slowing response. CBZ and CAL prevented aging phenotypes partially by ameliorating excitatory/inhibitory balance of excitable cells in aging worms via the transcription factor DAF‐16 and its downstream genes *numr‐1/−2*. Thus, we identify small molecules as potential drugs for preventing behavioral deterioration, and demonstrate that excitatory/inhibitory imbalance could be a target for promoting healthy aging (Figure 6e).

The neurotransmitter systems change greatly during aging, resulting in declines in cognitive abilities (Lee & Kim, 2022). Previously, through genome‐wide RNAi screening, we had successfully identified genes that regulate age‐related loss of 5‐HT and DA and behavioral deterioration (Yuan et al., 2020). In this study, using a similar strategy, we developed a reliable screening assay by which we successfully identified novel small molecules that slow down the aging process, demonstrating that pharmacological improvement of neurotransmitter function is an effective approach to promote healthy aging. Among 11 screening hits, Kobe0065 has been shown to repress the function of mTOR signal pathway, which regulates longevity (Blagosklonny, 2019). Hesperidin has been reported to have antioxidant activity that may protect against mitochondrial dysfunction and slow down aging (Chen et al., 2019; Fernando et al., 2016). However, the molecular links between the other compounds and aging remain to be further studied.

Electrical activity is critical for adult neuronal integrity. Disrupted neurotransmitter function or disturbed membrane excitability accelerates neuronal aging. At the system level, the inhibitory signaling decreases and the neural excitation increases with age. Preserving excitatory/inhibitory balance by inhibiting glutamatergic/cholinergic neuron activity or upregulating GABA signaling ameliorates aging phenotypes and increases longevity (Wirak et al., 2022; Zullo et al., 2019). The findings that CBZ and CAL suppress over‐excitability of diagonal muscles in aging males, agree with the notion that improved excitatory/inhibitory balance ameliorates behavioral decline in the elderly. CBZ is widely used in therapy of epilepsy by blocking Na^+^ influx into neuronal axon, causing inhibition of action potentials and synaptic transmission (Ambrosio et al., 1999; Willow & Catterall, 1982). In *C. elegans*, we found that CBZ influences cell excitability and the aging process by promoting Ca^2+^ homeostasis via inhibiting L‐type and P/Q‐type Ca^2+^ channels, demonstrating a novel mechanism underlying the function of CBZ. Interestingly, some other anticonvulsants, including Valproic acid, Ethosuximide and Trimethadione, have also been reported to regulate *C. elegans* lifespan by the similar mechanism (Evason et al., 2005, 2008).

The precise intracellular calcium homeostasis is essential for neuronal activity and function. Persistently elevated intracellular Ca^2+^ levels contribute to the plasticity deficits associated with aging process (Celsi et al., 2009; Siman et al., 1989). In Alzheimer's disease, the extracellular accumulation of amyloid‐β promotes Ca^2+^ influx into neurons, resulting in excitotoxic neurodegeneration, and blockage of calcium channels is neuroprotective (Nimmrich & Eckert, 2013). CAL is a complexometric indicator of calcium and magnesium (Rasouli & Ghavami, 2016), and it may help to maintain Ca^2+^ homeostasis and ameliorate excitation/inhibition balance in aging worms. This notion is supported by the findings that the function of CBZ and CAL on promoting healthy aging depends on NUMR‐1/−2, which mitigate stress‐induced damage (Bayer et al., 2005; Tvermoes et al., 2010).

In this study, we found that CBZ and CAL induced nuclear translocation of DAF‐16, the key transcription factor that regulates longevity. Similarly, previous studies have also shown that reduction in neuronal activity induced by genetic or pharmacological interventions delays aging via promoting nuclear translocation of DAF‐16 (Zullo et al., 2019). However, the mechanism how neuronal activity affect nuclear translocation of DAF‐16 remains elusive. One explanation is that these interventions ameliorate Ca^2+^ homeostasis, which in turn affect the activity of kinases or phosphatases, and finally affect the phosphorylation level as well as nuclear translocation of DAF‐16. Further studies on the mechanism underlying this phenomenon will provide useful clues to slow down aging and prevent age‐related diseases.

## 4 MATERIALS AND METHODS

### 4.1 Strains and culture of worms

Worms were fed with *Escherichia coli* OP50 and cultivated on standard NGM plates at 20°C unless stated otherwise. N2, TJ356, *tph‐1*(*mg280*), *cat‐2*(*e1112*), *daf‐16*(*mu86*), and *unc‐54*(*e190*) were obtained from the Caenorhabditis Genetics Center (CGC). The *acs‐20;P*_*bas‐1*_ *::bas‐1::gfp* transgenic worms were obtained by crossing *acs‐20* null mutant worms with *P*_*bas‐1*_ *::bas‐1::gfp* transgenic worms. The *P*_*unc‐68*_::*gcamp5/P*_*unc‐68*_::*dsred2* and *P*_*numr‐1/−2*_ *::numr‐1/−2* transgenic worms were generated by microinjection according to the methods described in the reference (Chen et al., 2015; Tvermoes et al., 2010).

### 4.2 Small‐molecule screen

Small‐molecule screen assays were performed as previously described (Jiang et al., 2018) with modifications. Chemical libraries including the MCE‐new Bioactive Compound Library, the Target Mol‐approved Drug Screening Library, and the AnalytiCon Discovery were used in this study. Synchronized L4 *acs‐20;P*_*bas‐1*_ *::bas‐1::gfp* worms were used for drug screening. The GFP intensity of NSM neurons in the transgenic worms was examined 9 days later by a Nikon A1R laser‐scanning confocal microscope.

### 4.3 Detection of 5‐HT and DA

5‐HT level of NSM neurons was detected using immunostaining, and DA level of CEP neurons was examined by formaldehyde‐induced fluorescence as previously described (Yin et al., 2014). The tested worms treated with DMSO, 1 μM CBZ, or 0.1 μM CAL. The fluorescent images of 5‐HT and DA were collected by a Nikon A1R laser‐scanning confocal microscope and an Olympus FV3000 confocal laser scanning microscope, respectively.

### 4.4 RNA interference

RNA interference by feeding was performed as previously described (Yin et al., 2014). L4 worms were transferred to NGM plates with HT115 bacteria expressing control dsRNAs or targeted gene‐specific dsRNAs. The empty L4440 plasmids were used as control dsRNAs. The primers used for RNA interference of *daf‐16* were, the forward primer AGT ACA GCA ATT CCC AAA TGA AA and the reverse primer AAT TGG ATT TCG AAG AAG TGG AT; Primers used for RNA interference of *numr‐1/−2* were, the forward primer TGA AAA CTA CAA CTG CAA C and the reverse primer TTA ACA TCG ACC AAA TCT GC; Vectors were confirmed by sequencing.

### 4.5 Behavioral assays

For pharyngeal pumping assays, we examined the number of pharyngeal contractions within 10 s intervals for each worm using a dissection microscope as described (Yin et al., 2014). Male mating assays were performed as previously described (Yin et al., 2014). The mating efficiency was shown as the percentage of plates that had successful mating. Two food‐induced slowing response behaviors, BSR and ESR, were performed as previously described (Yuan et al., 2020). The numbers of body bends within 20 s intervals were recorded under a dissection microscope.

Lifespan assays were performed as described (Yin et al., 2014). About 90 synchronized L4 worms were seeded on three NGM plates with 20 μM 5‐fluoro‐2′‐deoxyuridine (FUdR). Worms at day 2 of adulthood were transferred to new cultivating plates containing 20 μM FUdR. Dead worms were then counted every day.

Worm maximum velocity was measured as previously described (Hahm et al., 2015; Yin et al., 2017). 25 synchronized worms were selected from two OP50‐seeded NGM plates, and then were transferred to two new plates without bacterial lawns, where the maximum velocity of worms was recorded for 1 min at a rate of 10 frames per second by a stereomicroscope with an OLYMPUS DP72 CCD camera. Locomotion velocity was quantified by analyzing the first 300 frames of each recording using ImageJ and wrMTrck software. The peak velocity of locomotion was regarded as the maximum velocity.

Associative learning assays were performed as previously described (Kauffman et al., 2011). Synchronized worms were starved in M9 buffer for 1 h, then transferred to a 60‐mm NGM plate containing 500 μL OP50 and 2 μL 10% butanone for another hour. The chemotaxis to butanone of worms was calculated by Learning index = Chemotaxis index_Trained_−Chemotaxis index_Naïve_.

### 4.6 Stress assays

We performed hydrogen peroxide stress, ultraviolet stress, and heat‐shock assays as described previously (Yuan et al., 2020). Synchronized L4 worms were transferred to NGM plates containing 1 μM CBZ or 1 μM CAL or 0.1% DMSO. Worms at day 5 of adulthood were picked out for stress assays.

### 4.7 Nuclear localization of DAF‐16

TJ356 L4 larvae expressing DAF‐16::GFP fusion proteins were treated with 1 μM CBZ or 1 μM CAL in NGM plates. Two days later, the worms were then transferred to a 2% agarose pad on a glass slide, anesthetized by 50 μM NaN_3_, and imaged by a Nikon A1R laser‐scanning confocal microscope. The localization status of DAF‐16 was categorized as cytosolic localization when the fluorescence diffused in the cytoplasm, nuclear localization when DAF‐16 mainly existed in the nucleus throughout the entire body, or intermediate localization when DAF‐16 showed visible but not completely nuclear localization.

### 4.8 Calcium imaging

The activity of male worm diagonal muscles was monitored as described previously (Chen et al., 2015). 4 × objective lens together with 4 × zoom was used to obtain images of Ca^2+^ transient and the images were captured by a Nikon A1R laser scanning confocal microscope at 2 Hz. The value of GCaMP5/DsRed2 in diagonal muscles of free‐moving males (without hermaphrodite) was regarded as R0. ΔR/R0 in diagonal muscles at multiple time points was examined before and after turning. The time point when males' posterior tails reached the terminus of hermaphrodites' body (heads or tails) was regarded as 0 s.

### 4.9 Electrophysiology

HEK293T cells were cultured in DMEM medium (Gibco, 11,965,118) with 10% FBS (Gibco, 10,099,141) and 1% penicillin and streptomycin (Hyclone, SV30010) in 5% CO_2_ incubator at 37°C. Cells were transfected with plasmids of Cav1.2 channel subunits (α1 [0.2 μg], β3 [0.2 μg], α2δ [0.2 μg], and gfp [0.1 μg]) or Cav2.1 channel subunits (α1 [0.2 μg], β1_b_ [0.2 μg], α2δ [0.2 μg], and gfp [0.1 μg]) using the GP‐mate transfection reagent when cells were grown to 70% in 24‐well plates.

After 2 days of transfection, the HEK293T cells were used for whole‐cell recordings at room temperature. The extracellular solution contained 6 mM CaCl_2_, 140 mM TEA‐Cl, 5 mM MgCl_2_, 4 mM 4‐AP, and 10 mM HEPES. The intracellular solution consisted of 140 mM CsCl, 5 mM TEA‐Cl, 4 mM Mg‐ATP, 5 mM EGTA, and 5 mM TES. The pH of intracellular and extracellular solutions was adjusted to 7.2 with CsOH. The recordings were made using an EPC10 patch‐clamp amplifier (HEKA) and filtered at 1 kHz. The holding potential was −80 mV, and the Ca^2+^ currents were acquired by repolarizing voltage from −40 mV to +45 mV (in 5 mV increments). Data analysis was performed using Stimfit 0.15 and Igor Pro.

### 4.10 RNA isolation and RT‐PCR analysis

Total RNA was isolated by TRIzol Reagent (Invitrogen) from 600 to 700 synchronized worms at day 5 of adulthood. cDNAs were synthesized with the Reverse Transcriptase Kit (Qiagen) from 600 ng RNAs. Real‐time PCR assays were performed on a Roche LightCycler 480 system using SYBR Green (Takara). The *pmp‐3* gene was used as an internal reference. All procedures were performed following the manufacturer's protocols.

### 4.11 RNA‐seq and data analysis

About 600–700 synchronized L4 worms were transferred to NGM plates containing 1 μM CBZ or 1 μM CAL or 0.1% DMSO. Worms at day 5 of adulthood were collected for RNA‐seq assays. All experiments were performed in triplicate, and making up a total of nine samples. The RNA‐seq library were constructed followed by PCR amplified using Phusion DNA polymerase (NEB) for 15 PCR cycles. After quantified by Qubit 4.0, paired‐end RNA‐seq sequencing libraries were sequenced with the NovaSeq 6000 sequencer (2 × 150 bp read length). Gene expression values were normalized for sequencing depth with Fragments per Kilobase of Mapped reads (FPKM) and Transcripts per Million (TPM) methods. To identify DEGs between CBZ or CAL‐treated samples and corresponding control samples, the differential expression analysis was performed using the DESeq2. DEGs with |log2FC| > 1 and FDR < 0.05 were considered to be significantly different expressed genes.

## AUTHOR CONTRIBUTIONS

S.‐W. Y., S.‐D. S. and S.‐Q. C. designed the study; S.‐W. Y., S.‐D. S., Y. L. and X.‐J. L. performed experiments; S.‐W. Y., S.‐D. S. and S.‐Q. C. analyzed data; and S.‐W. Y. and S.‐Q. C. wrote the paper. S.‐W. Y. and S.‐D. S. contributed equally to this work.

## FUNDING INFORMATION

This work was supported by grants to S.‐Q. C. from the National Key R&D Program of China (2023YFC3603300 and 2023YFC3603400); and National Natural Science Foundation of China (grants 31,925,022 and 82,330,047).

## CONFLICT OF INTEREST STATEMENT

The authors declare no conflicts of interest.

## Supporting information


Figures S1–S8.

Tables S1–S5.

## Data Availability

All data supporting the findings of this study are included in this article and the supplementary materials. The data that support the findings of this study are available from the corresponding author upon reasonable request.
