# Supplementary material for: A small‐molecule screen identifies novel aging modulators by targeting 5‐HT/DA signaling pathway
Source: Aging Cell. 2024 Nov 18;24(3):e14411. doi: 10.1111/acel.14411 (PMC11896485; doi:10.1111/acel.14411)
Supplement: Supplementary file 1 — Figures S1–S8. [file ACEL-24-e14411-s001.pdf]

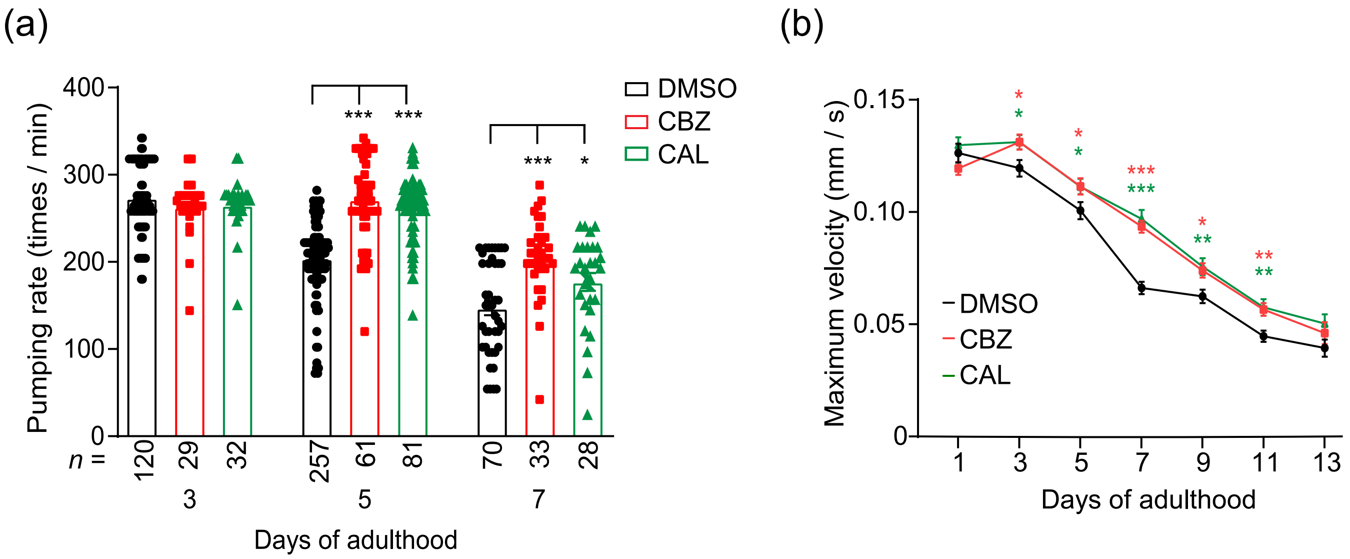

Figure S1. CBZ and CAL affect age-dependent decline in pharyngeal pumping and locomotion behaviors. Changes in pharyngeal pumping rate (a) and maximum velocity (b) in worms treat-ed with different concentrations of CBZ and CAL during aging. The numbers of tested worms are shown beneaththe bar. Data were from at least three independent experiments and data shown are means ± s.e.m.*P <0.05; **P < 0.01; ***P < 0.001; (a, one-way ANOVA test; b, two-sided log-rank test).

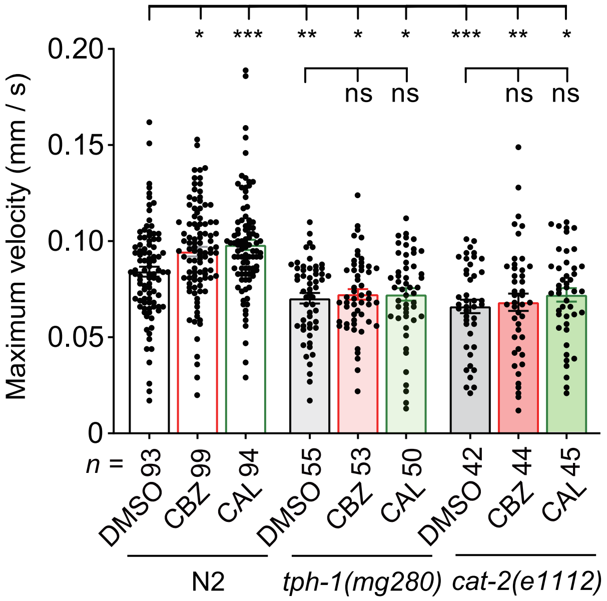

Figure S2. 5-HT and DA deficiency diminish the beneficial effects of CBZ and CAL on aging phenotypes. Age-dependent changes of maximum velocity of N2, *tph-1(mg280)* and *cat-2(e1112)* mutant worms treated with DMSO, CBZ, or CAL. The numbers of tested worms are shown beneath the bars. Data were from at least three independent experiments and data shown are means ± s.e.m. *$P < 0.05$; **$P < 0.01$; ***$P < 0.001$; ns, not significant (one-way ANOVA test).

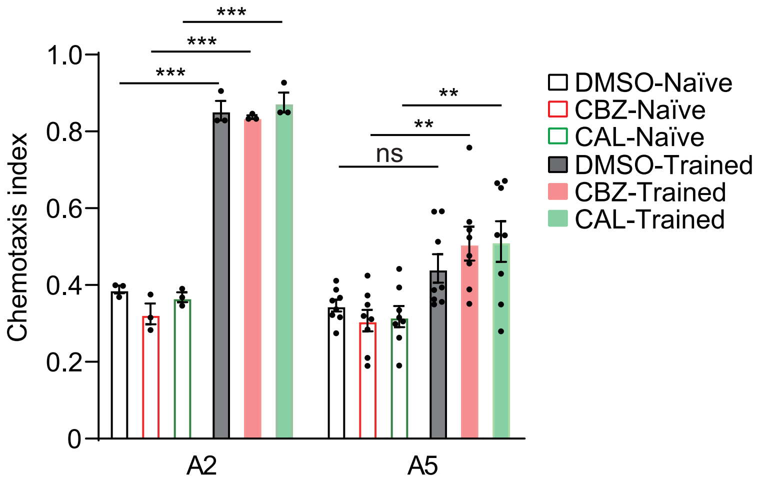

Figure S3. Chemotaxis index of DMSO-, CBZ- or CAL-treated worms at day 2 (A2) or day 5 (A5) ofadulthood. Data were from at least three independent experiments. ** $P < 0.01$; *** $P < 0.001$; ns,not significant (one-way ANOVA test).

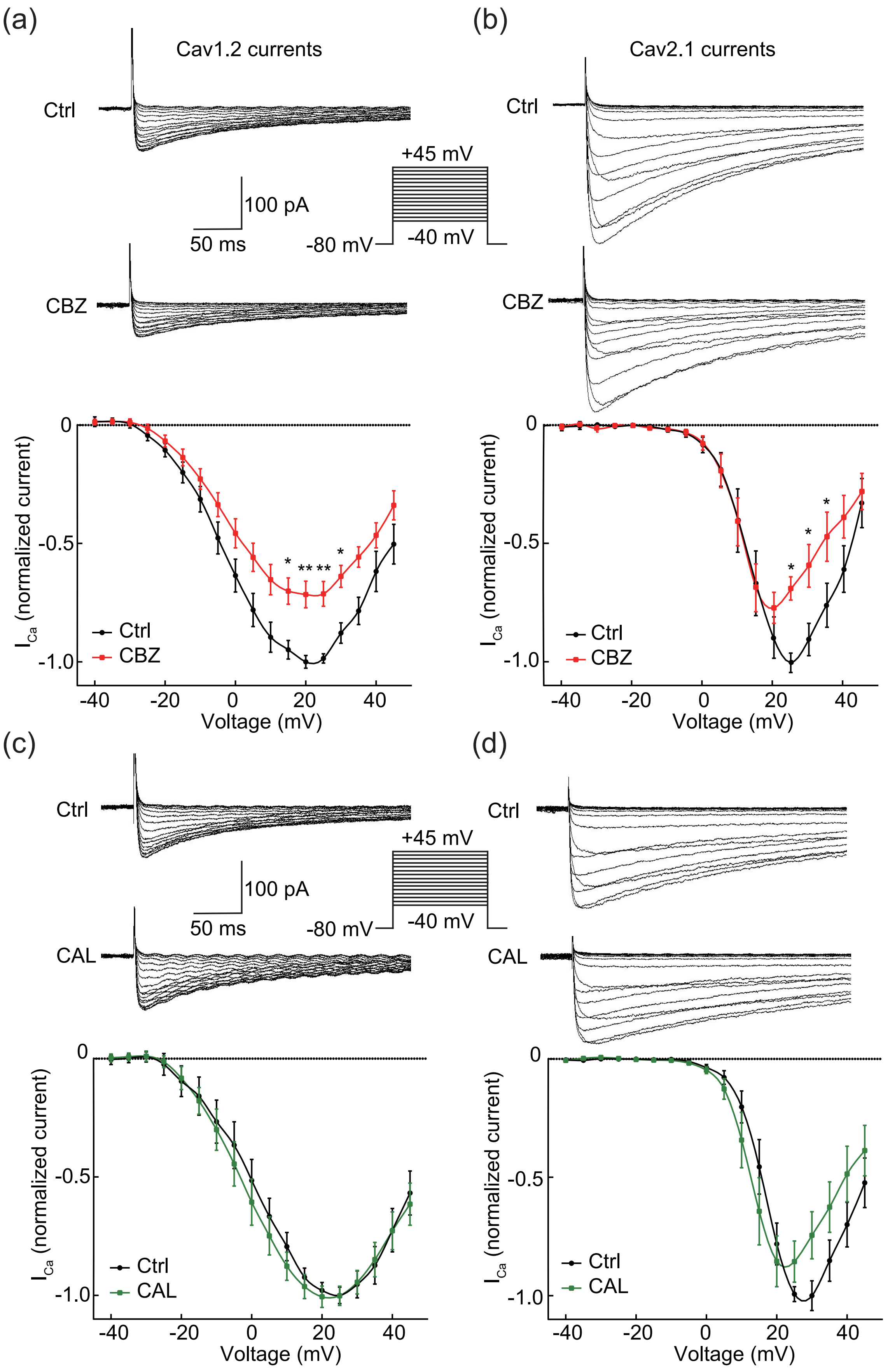

Figure S4. CBZ blocks Cav1.2 and Cav2.1 currents. (a) Representative whole-cell currents and current -to-voltage (I-V) relationship of Cav1.2 currents recorded in HEK293T cells before and after 1 μM CBZ (n= 11) perfusion. (b) Representative whole-cell currents and I-V relationship of Cav2.1currents recorded in HEK293T cells before and after 1 μM CBZ (n = 8) perfusion. (c, d) Representative whole-cell currentsand I-V relationship of Cav1.2 (n = 8) and Cav2.1 (n = 6) currents recorded in HEK293T cells before and after1 μM CAL perfusion. Data shown are means ± s.e.m. *P < 0.05; **P < 0.01 (two-wayANOVA test).

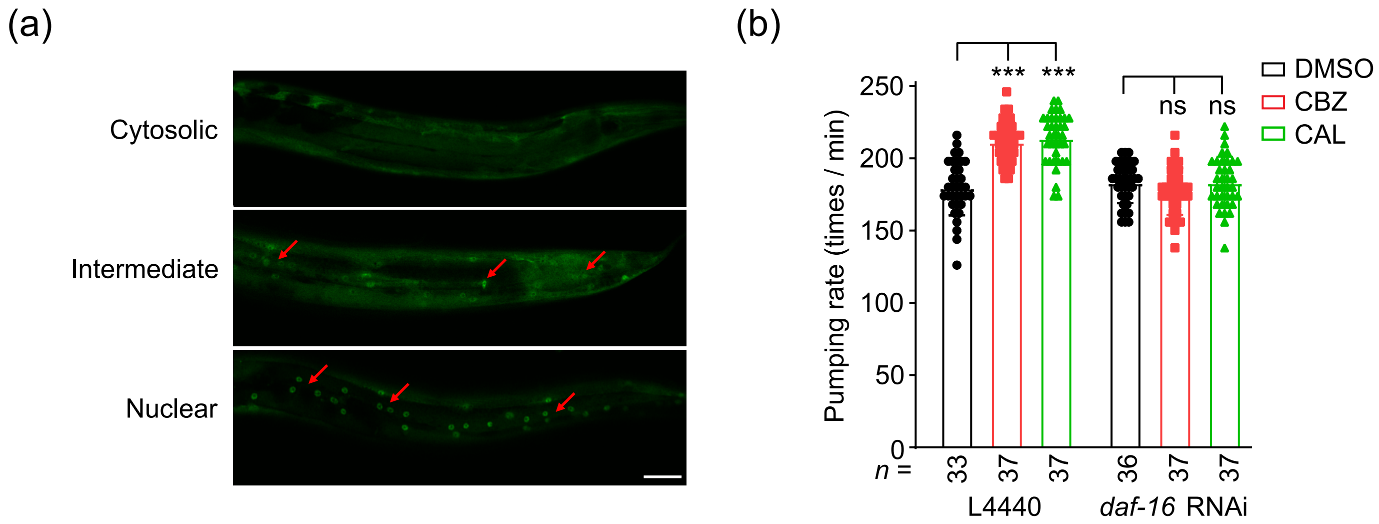

Figure S5. Knock-down of daf-16 abolishes CBZ or CAL's effect on pharyngeal pumping. (a) Three localiza-tion patterns of DAF-16::GFP: cytosolic (up; no nuclear GFP fluorescence), intermediate (middle; weaknuclear GFP fluorescence), and nuclear (down, strong nuclear GFP fluorescence). Scale bar, 20 µm. (b)Pharyngeal pumping rates of N2 worms fed with control or *daf-16* dsRNAs in the presence of DMSO, CBZor CAL. The numbers of tested worms are shown beneath the bars. Data were from at least three indepen-dent experiments. Data shown are mean ± s.e.m. ***$P < 0.001$; ns, not significant (one-way ANOVA test).

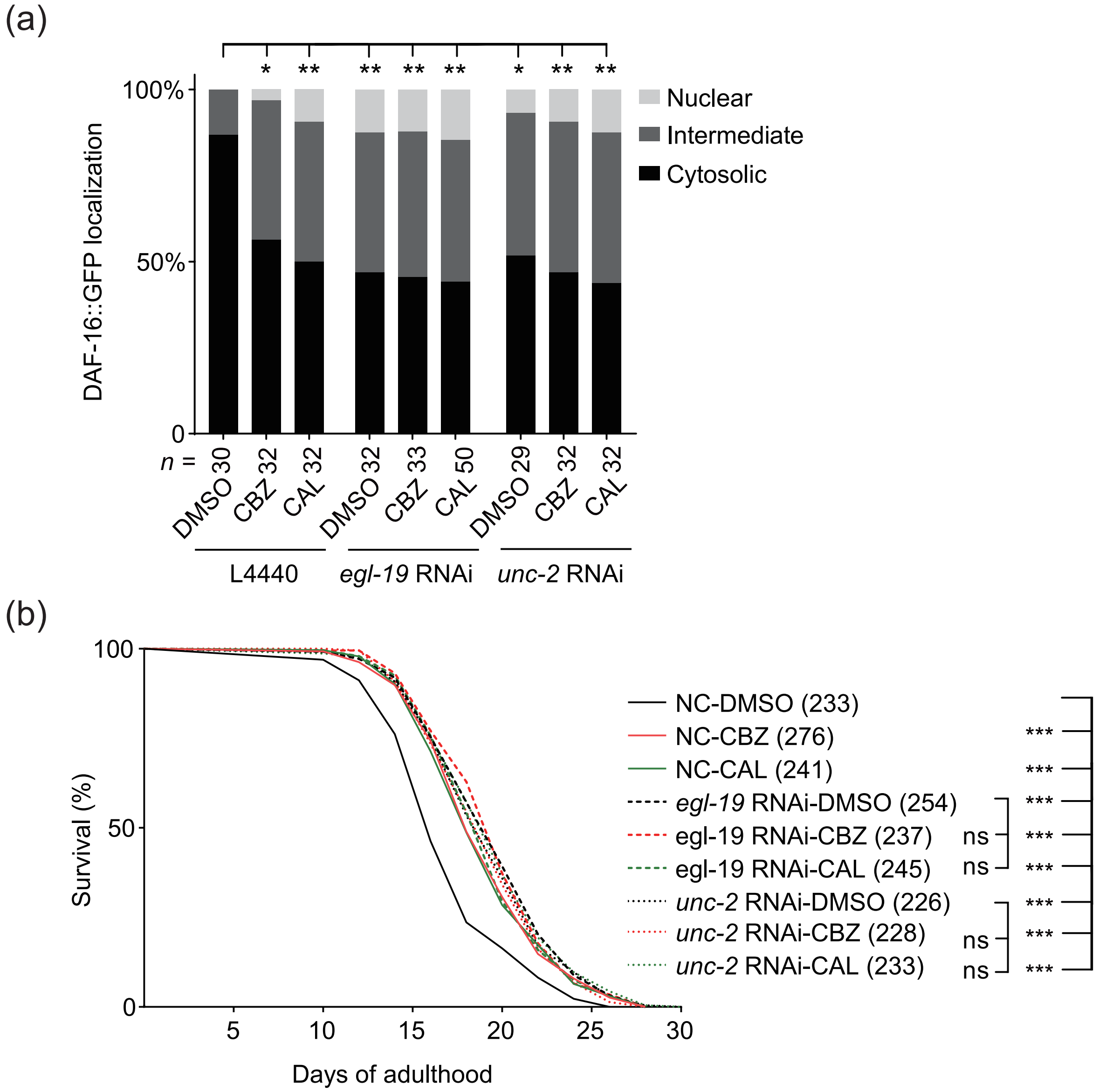

Figure S6. Knock-down of *egl-19* or *unc-2* abolishes CBZ or CAL's effect on DAF-16 nuclear translocation and the lifespan of worms. (a) Quantitative analysis of the cellular localization of DAF-16 in worms fed with HT115 bacteria expressing either control dsRNAs or specific dsRNAs targeting *egl-19* or *unc-2* genes in the presence of DMSO, CBZ, or CAL. The numbers of tested worms are shown beneath the bars. (b) Lifespan curves of worms treated with *egl-19* or *unc-2* dsRNAs in the presence of DMSO, CBZ, or CAL. Data represent the sum of animals in multiple experiments. Data were from at least three independent experiments. *$P < 0.05$; **$P < 0.01$; ***$P < 0.001$; ns, not significant (a, one-way ANOVA test; b, two-sided log-rank test).

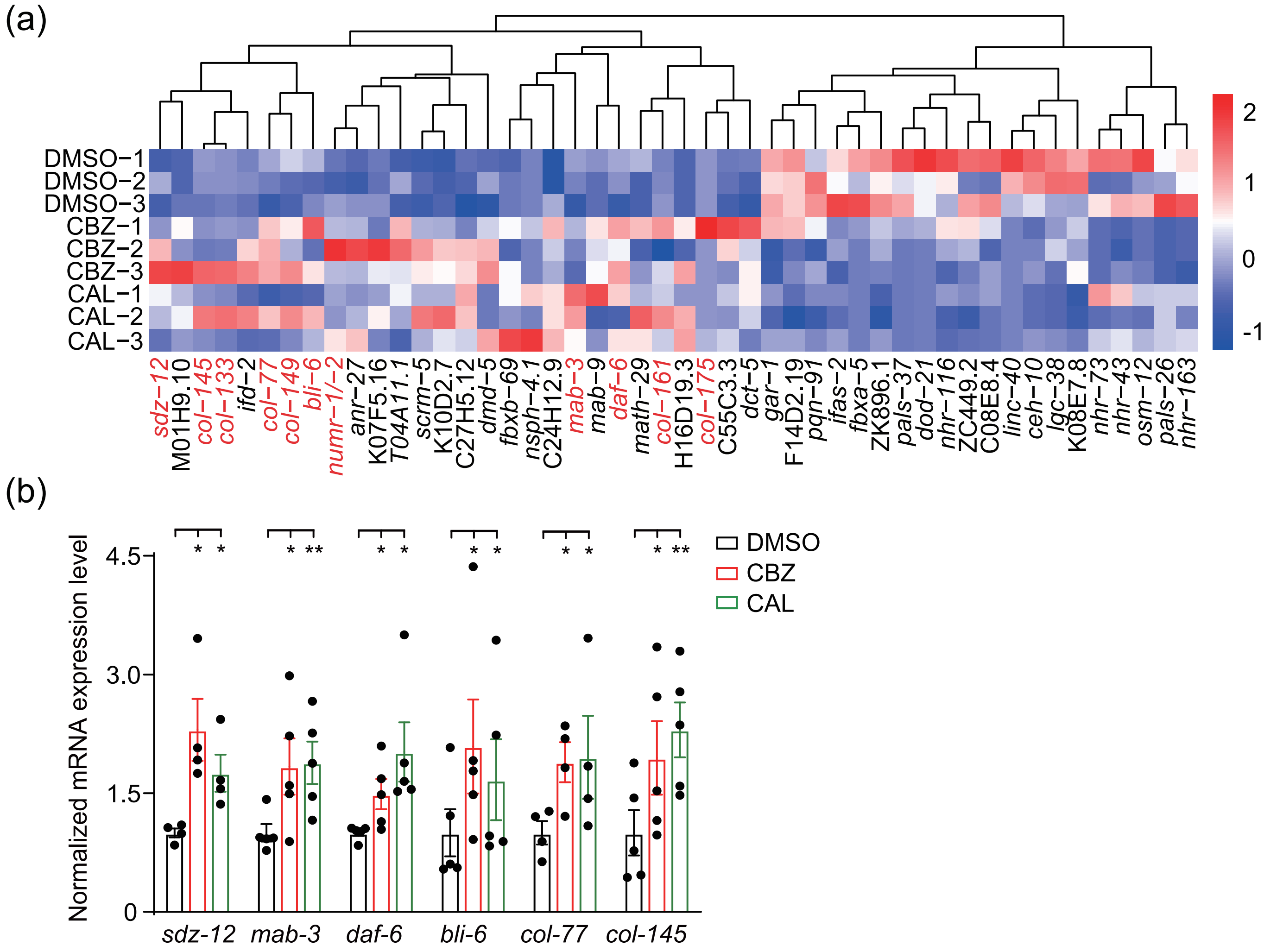

Figure S7. Administration of CBZ or CAL causes changes of gene expression in aging worms. (a) Heatmapshowing normalized expression of selected differentially expressed genes (DEGs) that are significantly changed byCBZ or CAL treatment compared with DMSO treatment. IIS pathway downstream genes are highlighted in red. (b)RT-PCR analysis of selected DAF-16 signaling-associated genes. The expression levels of these genes werenormalized with *pmp-3*. Data are shown as mean ± SEM. *P < 0.05; **P < 0.01 (one-way ANOVA test).

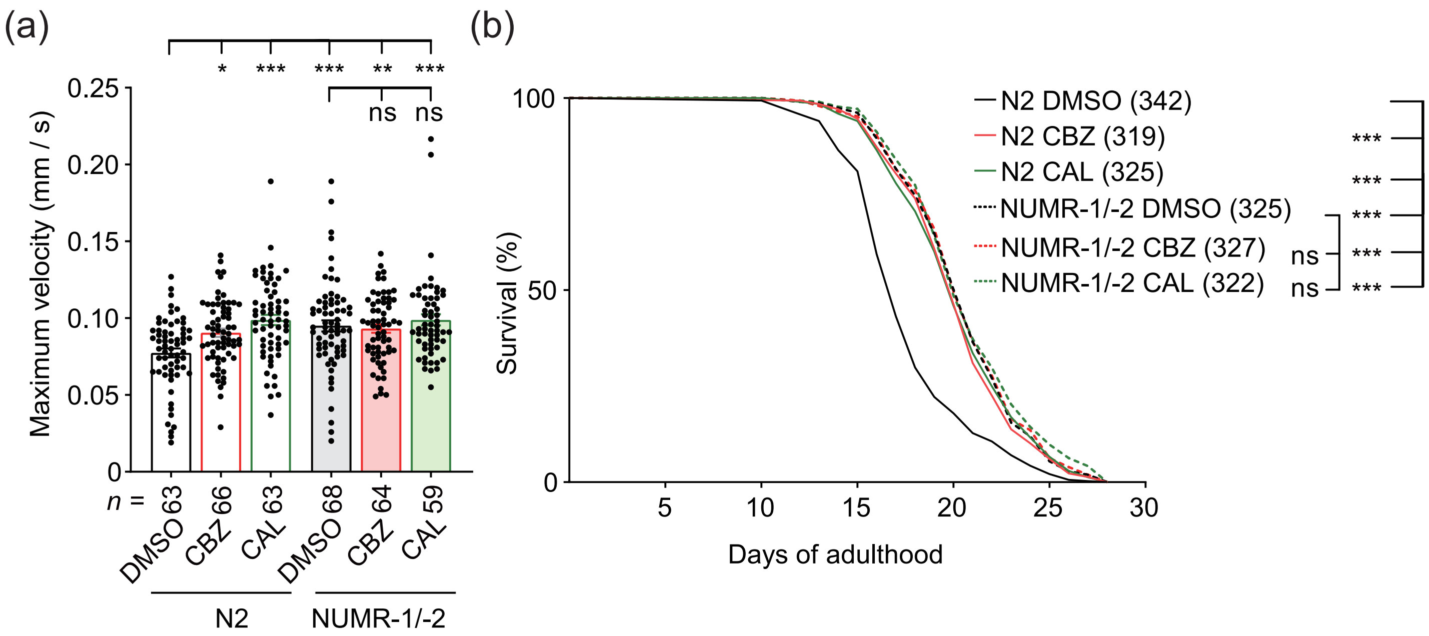

Figure S8. NUMR-1/-2 promotes healthy aging in *C. elegans*. (a) Age-dependent changes of maximum velocity of N2 and the transgenic worms expressing NUMR-1/-2 in the presence of DMSO, CBZ, or CAL. The numbers of tested worms are shown beneath the bars. (b) Lifespan curves for N2 and the transgenic worms in the presence of DMSO, CBZ, or CAL. Data represent the sum of animals in multiple experiments. Data were from at least three independent experiments. $^{*}P < 0.05$; $^{**}P < 0.01$; $^{***}P < 0.001$; ns, not significant (a, one-way ANOVA test; b, two-sided log-rank test).
