# Supplementary material for: A small‐molecule screen identifies novel aging modulators by targeting 5‐HT/DA signaling pathway
Source: Aging Cell. 2024 Nov 18;24(3):e14411. doi: 10.1111/acel.14411 (PMC11896485; doi:10.1111/acel.14411)
Supplement: Supplementary file 2 — Tables S1–S5. [file ACEL-24-e14411-s002.docx]

**Table S1**

Three compound libraries used in the small-molecule screen.

| Compound Library | Type | Count | Total |
| --- | --- | --- | --- |
| MCE-new bioactive compound library (M) | FDA approved | 4453 | 10459 |
| Target Mol-approved drug screening library (T) | FDA or EMA approved | 1600 |  |
| AnalytiCon Discovery 4406 (A) | Plant and microbial extracts | 4406 |  |

**Table S2**

55 compounds up-regulated the level of BAS-1::GFP at a *P* value less than 0.01.

| Compound | Description | Bioactivity | *P* value |
| --- | --- | --- | --- |
| Dacomitinib (M2-G9) | Dacomitinib is a specific and irreversible inhibitor of the ERBB family | antitumor activity | < 0.0001 |
| Veliparib (M31-B3) | Veliparib is a potent inhibitor of PARPs | antitumor activity | 0.0022 |
| Silmitasertib (M50-E10) | Silmitasertib is a highly selective and potent CK2 inhibitor | antitumor activity | 0.0016 |
| Kobe0065 (M51-B7) | Kobe0065 is an inhibitor of Ras-Raf interaction | antitumor activity | 0.0016 |
| Gallic acid (M14-E4) | Gallic acid is a free radical scavenger with antimicrobial activity | anti-inflammatory, antitumor activity | 0.0067 |
| Kresatin (T14-G11) | Kresatin is a pharmaceutical intermediate and antiseptic | anti-inflammatory activity | 0.0004 |
| Sarafloxacin (T12-E4) | Sarafloxacin is a fluoroquinolone antibiotic registered | anti-inflammatory activity | 0.0016 |
| Hesperidin (M50-D9) | Hesperidin is an anti-inflammatory agent and exerts antioxidant effects | anti-inflammatory, antioxidant activity | 0.008 |
| Rosmarinic acid (M50-B4) | Rosmarinic acid exerts a cytoprotective effect by scavenging intracellular ROS | antioxidant activity | 0.0012 |
| Carbamazepine (M13-F9) | Carbamazepine is a sodium channel blocker and used to prevent seizures | regulating cell excitability | 0.0032 |
| Calmagite (M50-A4) | Calmagite is a complexometric indicator for detecting calcium and magnesium | regulating cell excitability | 0.0011 |
| L-Ascorbic acid (M12-A5) | L-Ascorbic acid is an effective reducing agent and donor antioxidat | antioxidant activity | 0.0073 |
| Roxatidine (M14-H6) | Roxatidine can suppress inflammatory response and have antitumor activity | anti-inflammatory, antitumor activity | 0.0022 |
|  |  |  |  |
| Cefixime (M15-G2) | Cefixime is an antibiotic and a third-generation cephalosporin antibiotic | anti-inflammatory | 0.0083 |
| Sotrastaurin (M30-D3) | Sotrastaurin is a potent pan-PKC inhibitor | pan-PKC inhibitor | 0.0092 |
| ICI 118,551 (M30-G3) | ICI 118,551 is a highly selective β2 adrenergic receptor antagonist | β2 adrenergic receptor antagonist | 0.0085 |
| AT7519 (M31-F3) | AT7519 Hydrochloride is a potent inhibitor of CDKs | antitumor activity | 0.0074 |
| Targocil (M34-B2) | Targocil functions as a bacteriostatic inhibitor of wall teichoic acid biosynthesis | anti-inflammatory | 0.0047 |
| Gynostemma Extract (M35-G6) | Gynostemma Extract is a natural product. | N/A | 0.0064 |
| WAY-100635 Maleate (M35-H6) | WAY-100635 Maleate is a potent and selective 5-HT1A receptor antagonist | 5-HT1A receptor antagonist | 0.004 |
| ML204 (M37-D4) | ML204 is an antagonist that selectively modulates native TRPC4/C5 channels | regulating cell excitability | <0.0001 |
| ML-098 (M42-H4) | ML-098 is an activator of the GTP-binding protein Rab7. | neuroprotective activity | 0.0026 |
| ESI-09 (M43-E6) | ESI-09 is a novel noncyclic nucleotide EPACs antagonist | anti-inflammatory, antitumor activity | 0.002 |
| N6022 (M44-H11) | N6022 is a potent S-Nitrosoglutathione reductase (GSNOR) inhibitor | regulating NO | 0.0036 |
| KN-62 (M52-F4) | KN-62 is a selective and potent inhibitor of calmodulin-dependent protein kinase II | inhibiting CaMK-II | 0.0077 |
| ZM-447439 (M52-E6) | ZM-447439 is an aurora kinase inhibitor | antitumor activity | 0.002 |
| Lorlatinib (M54-H11) | Lorlatinib is a potent, dual ALK/ROS1 inhibitor | antitumor activity, antioxidant activity | 0.0033 |
| Mericitabine (M55-D3) | Mericitabine is a nucleoside inhibitor of the HCV NS5B polymerase | antiviral activity | 0.0086 |
| PF-01247324 (M56-H3) | PF-01247324 is a selective Nav1.8 blocker | regulating cell excitability | 0.0038 |
| Lomustine (M13-B11) | Lomustine is a DNA alkylating agent, with antitumor activity | antitumor activity | 0.0006 |
| Ivosidenib (M28-F2) | Ivosidenib is a mutant isocitrate dehydrogenase1 inhibitor | antitumor activity | 0.0073 |
| Atractylenolide III (M31-A4) | Atractylenolide III is a major component of Atractylodes rhizome can induce apoptosis | antitumor activity | <0.0001 |
| GSK269962A (M49-G11) | GSK269962A is a potent ROCK inhibitor | anti-inflammatory and vasodilatory activities | <0.0001 |
| CCR2-RA-[R] (M50-A8) | CCR2-RA-[R] is an allosteric antagonist of the CCR2 | anti-inflammatory | 0.0006 |
| Guaiacol (T14-G10) | Guaiaco inhibits LPS-stimulated COX-2 expression and NF-κB activation | anti-inflammatory | 0.0007 |
| A53-C8 | From a microbial and plant derived pure natural products library | N/A | < 0.0001 |
| A53-F9 | From a microbial and plant derived pure natural products library | N/A | < 0.0001 |
| A34-E3 | From a microbial and plant derived pure natural products library | N/A | < 0.0001 |
| A41-E11 | From a microbial and plant derived pure natural products library | N/A | < 0.0001 |
| A41-H11 | From a microbial and plant derived pure natural products library | N/A | < 0.0001 |
| A54-D6 | From a microbial and plant derived pure natural products library | N/A | < 0.0001 |
| A10-A4 | From a microbial and plant derived pure natural products library | N/A | 0.0008 |
| A10-F7 | From a microbial and plant derived pure natural products library | N/A | < 0.0001 |
| A11-D8 | From a microbial and plant derived pure natural products library | N/A | 0.0003 |
| A18-F8 | From a microbial and plant derived pure natural products library | N/A | 0.0004 |
| A19-C11 | From a microbial and plant derived pure natural products library | N/A | 0.0009 |
| A46-C5 | From a microbial and plant derived pure natural products library | N/A | 0.0007 |
| A54-C4 | From a microbial and plant derived pure natural products library | N/A | 0.0001 |
| A10-D4 | From a microbial and plant derived pure natural products library | N/A | 0.0064 |
| A28-E8 | From a microbial and plant derived pure natural products library | N/A | 0.0089 |
| A35-C5 | From a microbial and plant derived pure natural products library | N/A | 0.0011 |
| A33-H7 | From a microbial and plant derived pure natural products library | N/A | 0.0031 |
| A33-H8 | From a microbial and plant derived pure natural products library | N/A | 0.0046 |
| A36-H3 | From a microbial and plant derived pure natural products library | N/A | 0.0094 |
| A46-G3 | From a microbial and plant derived pure natural products library | N/A | 0.0032 |

**Table S3**

CBZ or CAL induced gene expression changes assessed by RNA sequencing.

| CBZ vs DMSO | | | |
| --- | --- | --- | --- |
| Gene name | Gene description | FC | FDR |
| *col-175* | Putative cuticle collagen | 49.27 | 4.17E-02 |
| *clec-247* | C-type lectin domain-containing protein | 41.29 | 1.93E-03 |
| *mai-1* | ATPase inhibitor mai-1 | 38.38 | 1.73E-02 |
| *nlp-32* | YGGWG-amide | 25.70 | 1.90E-02 |
| *math-7* | MATH domain containing | 24.91 | 1.21E-02 |
| *str-31* | Seven TM receptor | 23.05 | 1.86E-02 |
| *fipr-10* | Fungus-induced protein | 21.68 | 2.54E-02 |
| *prmt-4* | Methyltransf 25 domain-containing protein | 18.02 | 4.10E-02 |
| *scrm-5* | Phospholipid scramblase | 17.59 | 1.17E-02 |
| C09F12.3 | GPCR domain-containing protein | 14.25 | 3.04E-02 |
| *sri-16* | Serpentine receptor, class I | 13.07 | 3.61E-02 |
| *grd-17* | Hedgehog-like protein | 12.81 | 4.09E-02 |
| K07F5.16 | J domain-containing protein | 12.15 | 9.62E-03 |
| *sdz-12* | Zinc finger protein | 10.36 | 1.61E-02 |
| *col-133* | Putative cuticle collagen | 10.10 | 4.86E-02 |
| *dct-5* | Membrane protein | 7.33 | 2.49E-02 |
| F47B8.4 | Glutaredoxin domain-containing protein | 6.18 | 5.69E-03 |
| *ncs-7* | Neuronal Calcium Sensor family | 5.75 | 4.29E-02 |
| *nhr-235* | NR LBD domain-containing protein | 5.10 | 3.75E-02 |
| *ilys-2* | Invertebrate-type lysozyme 2 | 4.66 | 1.34E-02 |
| *ifd-2* | Intermediate filament protein | 4.50 | 6.13E-04 |
| K06A9.3 | EGF-like domain-containing protein | 4.49 | 3.29E-02 |
| F29A7.4 | CB1 cannabinoid receptor-interacting protein | 4.31 | 3.25E-02 |
| F18A11.5 | BTB domain-containing protein | 4.24 | 3.03E-02 |
| *mab-9* | T-box protein 12 | 4.23 | 1.64E-02 |
| *npr-35* | GPCR domain-containing protein | 3.69 | 4.06E-03 |
| *dmd-5* | DM domain-containing protein | 3.64 | 3.81E-02 |
| *bli-6* | Putative cuticle collagen | 3.33 | 2.20E-02 |
| *lgc-53* | Ligand-Gated ion Channel | 3.15 | 3.06E-02 |
| *gst-40* | Glutathione S-Transferase | 3.08 | 3.96E-02 |
| K10D2.7 | Molybdopterin synthase sulfur carrier subunit | 2.96 | 4.38E-02 |
| *ets-5* | ETS domain-containing protein | 2.92 | 1.24E-02 |
| *daf-6* | SSD domain-containing protein | 2.77 | 3.69E-02 |
| *flp-33* | FMRF-Like Peptide | 2.67 | 2.21E-02 |
| B0365.9 | CPG4 domain-containing protein | 2.60 | 2.78E-02 |
| *comt-5* | Catechol-O-MethylTransferase family | 2.45 | 4.44E-02 |
| *col-77* | Putative cuticle collagen | 2.39 | 2.14E-03 |
| *col-149* | Putative cuticle collagen | 2.36 | 2.06E-02 |
| *numr-2* | NUclear localized Metal Responsive | 2.35 | 3.79E-02 |
| *numr-1* | NUclear localized Metal Responsive | 2.35 | 3.79E-02 |
| *fbxb-52* | F-box domain-containing protein | 2.28 | 1.92E-02 |
| *nduo-2* | NADH-ubiquinone oxidoreductase chain 2 | 2.28 | 3.31E-02 |
| *bbln-1* | Bulges Budding from the intestinal LumeN | 2.25 | 1.20E-02 |
| *parg-2* | Poly(ADP-ribose) glycohydrolase 2 | 2.20 | 3.84E-02 |
| *bath-45* | BTB and MATH domain-containing protein 45 | 2.19 | 3.20E-02 |
| F59E12.15 | Protein kinase domain-containing protein | 2.14 | 1.57E-02 |
| *abu-10* | Activated in blocked unfolded protein response | 2.11 | 4.29E-02 |
| T16G1.7 | CHK domain-containing protein | 2.11 | 5.96E-03 |
| T08D2.8 | TOG domain-containing protein | 0.02 | 3.80E-02 |
| Y73B3B.1 | CH domain-containing protein | 0.03 | 3.68E-03 |
| *klp-11* | Kinesin-like protein | 0.04 | 2.61E-03 |
| *nhr-73* | Nuclear hormone receptor family | 0.04 | 2.83E-02 |
| *pals-26* | Protein containing ALS2cr12 signature | 0.05 | 1.78E-02 |
| T11F8.4 | Protein kinase domain-containing protein | 0.06 | 2.12E-02 |
| *spch-2* | SPerm CHromatin enriched | 0.06 | 3.82E-02 |
| F36H5.14 | MATH domain-containing protein | 0.06 | 4.68E-02 |
| *fbxa-5* | F-box domain-containing protein | 0.08 | 3.19E-02 |
| *pals-37* | Protein containing ALS2cr12signature | 0.10 | 2.31E-02 |
| Y70G10A.2 | EGF-like domain-containing protein | 0.14 | 9.59E-03 |
| *tsp-2* | TetraSPanin family | 0.15 | 2.15E-02 |
| *ddn-1* | Downstream of Daf-Nineteen | 0.20 | 2.71E-03 |
| *osm-12* | Bardet-Biedl syndrome 7 protein homolog | 0.22 | 1.69E-02 |
| W03A5.1 | Protein kinase domain-containing protein | 0.23 | 8.93E-03 |
| *ceh-10* | Homeobox protein | 0.23 | 3.29E-02 |
| *nhr-163* | Nuclear receptor domain-containing protein | 0.26 | 3.45E-03 |
| *xtr-1* | MX region of TRA-2 Related | 0.26 | 3.43E-02 |
| *tbx-40* | Putative T-box protein 40 | 0.27 | 3.79E-02 |
| *col-146* | Putative cuticle collagen | 0.30 | 3.98E-02 |
| *unc-105* | Derin-like protein unc-105 | 0.36 | 1.78E-02 |
| *ugt-44* | Glucuronosyltransferase | 0.42 | 7.84E-03 |
| *fbxb-98* | F-box domain-containing protein | 0.42 | 3.57E-02 |
| *catp-3* | Cation ATPase N domain-containing protein | 0.43 | 5.64E-03 |
| *ugt-65* | UDP-glucuronosyltransferase | 0.43 | 1.76E-02 |
| *nhr-43* | Nuclear hormone receptor family | 0.44 | 3.03E-02 |
| T05E11.8 | DUF148 domain-containing protein | 0.45 | 3.66E-02 |
| *sul-1* | Putative extracellular sulfatase Sulf-1 homolog | 0.47 | 4.09E-02 |
| *fbxa-98* | F-box domain-containing protein | 0.47 | 3.79E-02 |
| *ddr-1* | Discoidin domain-containing receptor A | 0.47 | 3.28E-02 |
|  | | | |
| CAL vs DMSO | | | |
| Gene name | Gene description | FC | FDR |
| *try-8* | Peptidase S1 domain-containing protein | 60.20 | 1.06E-03 |
| *ins-31* | INSulin related protein | 46.21 | 7.16E-03 |
| *gst-25* | GST class-pi | 29.72 | 9.52E-07 |
| K11D12.6 | BPTI/Kunitz inhibitor domain-containing protein | 29.46 | 2.74E-02 |
| *clec-134* | CW domain-containing protein | 25.77 | 3.91E-02 |
| *nsph-4.1* | Nematode Specific Peptide family | 23.89 | 4.87E-02 |
| H16D19.3 | C2H2-type domain-containing protein | 23.03 | 1.87E-02 |
| *scl-15* | SCP domain-containing protein | 21.02 | 2.18E-02 |
| *scl-8* | SCP domain-containing protein | 20.26 | 3.82E-03 |
| *srd-49* | GPCR domain-containing protein | 16.51 | 3.83E-02 |
| *nep-7* | Thermolysin-like zinc metallopeptidases | 16.03 | 4.31E-02 |
| *scrm-5* | Phospholipid scramblase | 15.53 | 1.77E-02 |
| *ceh-17* | Homeobox protein | 11.83 | 3.37E-02 |
| Y39A3B.1 | Peptidase M28 domain-containing protein | 11.81 | 3.60E-02 |
| *sre-36* | Serpentine Receptor, class epsilon | 10.89 | 4.70E-02 |
| C49G7.13 | CUB 2 domain-containing protein | 9.04 | 3.59E-02 |
| *ttr-22* | TransThyretin-Related family domain | 8.21 | 3.72E-02 |
| *math-29* | MATH domain containing | 7.55 | 4.47E-02 |
| *pud-1.1* | PUD1_2 domain-containing protein | 4.76 | 1.77E-02 |
| ZC239.5 | BTB domain-containing protein | 4.52 | 2.90E-03 |
| *col-145* | Putative cuticle collagen | 4.36 | 4.35E-02 |
| *fbxb-69* | FBA 2 domain-containing protein | 3.70 | 1.47E-02 |
| *mab-3* | Protein male abnormal 3 | 3.64 | 2.63E-02 |
| *sulp-1* | STAS domain-containing protein | 3.27 | 2.50E-02 |
| K10D2.7 | Molybdopterin synthase sulfur carrier subunit | 3.03 | 3.93E-02 |
| M03A1.8 | Cytochrome b561 domain-containing protein | 2.68 | 2.58E-03 |
| *grsp-1* | Glycine rich secreted protein | 2.18 | 3.68E-02 |
| *col-54* | Putative cuticle collagen | 2.13 | 1.23E-02 |
| *fbxb-34* | F-box domain-containing protein | 2.12 | 5.28E-03 |
| *col-161* | Putative cuticle collagen | 2.04 | 4.92E-02 |
| *pals-37* | Protein containing ALS2cr12 signature | 0.03 | 7.99E-04 |
| *mlc-7* | Myosin light chain | 0.03 | 2.91E-02 |
| *col-64* | Putative cuticle collagen | 0.04 | 2.25E-02 |
| C32H11.6 | DUF148 domain-containing protein | 0.06 | 4.11E-02 |
| *nspg-4* | Nematode specific peptide family | 0.06 | 3.90E-02 |
| *dod-21* | CUB 2 domain-containing protein | 0.07 | 4.54E-02 |
| *fbxa-66* | F-box domain-containing protein | 0.09 | 4.77E-02 |
| C49H3.16 | BPTI/Kunitz inhibitor domain-containing protein | 0.10 | 2.12E-02 |
| *sre-23* | Serpentine receptor, class epsilon | 0.17 | 4.34E-02 |
| *ceh-10* | Homeobox protein | 0.17 | 1.04E-02 |
| *pqn-91* | Prion-like-(Q/N-rich)-domain-bearing protein | 0.19 | 4.50E-02 |
| *ifas-2* | Inducible FAScin domain containing | 0.25 | 3.97E-02 |
| B0432.9 | Lactamase B domain-containing protein | 0.27 | 3.84E-02 |
| ZK896.1 | CUB 2 domain-containing protein | 0.29 | 2.25E-02 |
| *lgc-38* | Ligand-gated ion channel | 0.29 | 1.73E-02 |
| F18A11.2 | CRAL-TRIO domain-containing protein | 0.32 | 1.92E-02 |
| *gar-1* | Putative muscarinic acetylcholine receptor | 0.43 | 3.62E-02 |
| C08E8.4 | NADAR domain-containing protein | 0.43 | 3.28E-02 |
| *nhr-116* | Nuclear hormone receptor family | 0.48 | 3.24E-02 |
| *sox-3* | Transcription factor | 0.48 | 4.90E-02 |
| Genes with false discovery rate (FDR) corrected *p*-value less than 0.05 and fold-change (FC) in expression greater than 2 (red shading, up-regulated gene) or less than -2 (green shading, down-regulated gene) were shown in this table, encoding ncRNA and uncharacterized protein were excluded. | | | |

**Table S4**

The primers used in the RT-PCR assays.

| Target gene | Primer sequence (5’-3’) | |
| --- | --- | --- |
|  | Forward | Reverse |
| *mab-3* | GTACTCTTGTGGAACACCGTCG | GACCATCTGTAGCTGTGACAACC |
| *daf-6* | GCTGAGTCAACTTGGCGTCG | GATCTATGCCTGAGCTACGATTGG |
| *sdz-12* | CTTCAAAGGCACCTGCACAAC | GTGTACCAGGAGATGCCTAGG |
| *bli-6* | CTACGTCTCGCACGTGAAGAAG | CGTCCTGGATTTCCAGGAAGTC |
| *col-77* | CTTTGACTGCCATCGTCGCTAT | GCTTGACGCTTCACAAGCTTC |
| *col-145* | GGAGTCTCTGTTTTCCGTGTTGAG | GTGGGCAAGTTGGCTTAGTTGG |
| *numr-1/-2* | GGAAGACGAGGAATGGATCATC | CTGCGTTCAAATGGATGTTGTCTC |
| *pmp-3* | GTTCCCGTGTTCATCACTCAT | ACACCGTCGAGAAGCTGTAGA |

**Table S5**

The primers used for RNA interference.

| Target gene | Primer sequence (5’-3’) | |
| --- | --- | --- |
|  | Forward | Reverse |
| *daf-16* | AGTACAGCAATTCCCAAATGAAA | AATTGGATTTCGAAGAAGTGGAT |
| *cca-1* | CGCTGCTGGAGTGTTTGTAA | TCGACGGAGTGAAGTTTGTG |
| *egl-19* | GATATCCTCGTCGTTGCAGTATC | AAAAGTGTTCGAATTCCTTCTCC |
| *unc-2* | TGGAACAACCAAGTCAACCA | GCGGACAATAATCCAGTCGT |
| *unc-8* | AATCCCTTTGGTTTGATTTTTGT | GGAGGAGAGATGCTCACTGTAGA |
| *unc-77* | CAAAGTCTTCGAGAATCGGC | GAATGTCCTGCTCCAATGGT |
| *numr-1/-2* | TGAAAACTACAACTGCAAC | TTAACATCGACCAAATCTGC |
